# Detection and analysis of long noncoding RNA expression profiles related to epithelial–mesenchymal transition in keloids

**DOI:** 10.1186/s12938-022-00976-x

**Published:** 2022-01-11

**Authors:** Zhixiong Chen, Xi Chu, Jinghong Xu

**Affiliations:** 1 Department of Plastic Surgery, The First Affiliated Hospital, Zhejiang University School of Medicine, Hangzhou, 310003 People’s Republic of China; 2 Zhejiang University School of Medicine, Hangzhou, 310000 People’s Republic of China

**Keywords:** Keloid, Long noncoding RNA, Epithelial-mesenchymal transition

## Abstract

**Background:**

The role of epithelial-mesenchymal transition (EMT) in the pathogenesis of keloids is currently raising increasing attention. Long noncoding RNAs (lncRNAs) govern a variety of biological processes, such as EMT, and their dysregulation is involved in many diseases including keloid disease. The aim of this study was to identify differentially expressed EMT-related lncRNAs in keloid tissues versus normal tissues and to interpret their functions.

**Results:**

Eleven lncRNAs and 16 mRNAs associated with EMT were identified to have differential expression between keloid and normal skin tissues (fold change > 1.5, *P* < 0.05). Gene Ontology (GO) analysis showed that these differentially expressed mRNAs functioned in the extracellular matrix, protein binding, the positive regulation of cellular processes, the Set1C/COMPASS complex and histone acetyltransferase activity. Kyoto Encyclopedia of Genes and Genomes (KEGG) analysis demonstrated that these mRNAs are involved in pathways in cancer. The lncRNA, XLOC_000587 may promote cell proliferation and migration by enhancing the expression of ENAH, while AF268386 may facilitate the invasive growth of keloids by upregulating DDR2.

**Conclusions:**

We characterized the differential expression profiles of EMT-related lncRNAs and mRNAs in keloids, which may contribute to preventing the occurrence and development of keloids by targeting the corresponding signaling pathways. These lncRNAs and mRNAs may provide biomarkers for keloid diagnosis and serve as potential targets for the treatment of this disease.

**Supplementary Information:**

The online version contains supplementary material available at 10.1186/s12938-022-00976-x.

## Background

Keloids are benign lesions with invasive growth beyond the original wound borders, in which the hyperproliferation of fibroblasts and excessive accumulation of extracellular matrix can be observed [1]. The claw-like extension of keloids not only causes cosmetic disfigurement, but also brings about uncomfortable feelings such as itch and pain, as well as local dysfunction, resulting in impaired emotional wellbeing and reduced quality of life. However, the exact mechanism of keloid scarring remains unknown. Any disorder during wound healing, including an enhanced inflammatory response, overexpression of growth-related factors, and excessive activation of fibroblasts, is conducive to abnormal collagen deposition in keloids [2]. Transforming growth factor-β (TGF-β), connective tissue growth factor (CTGF), vascular endothelial growth factor (VEGF) and the renin–angiotensin system were reported to be potential targets in keloid management considering their participation in wound healing and keloid formation [3, 4]. In addition, miRNA modulation may be a novel therapeutic solution [5]. However, neither first-line therapies nor developing treatments could guarantee satisfying curative effects, which calls for more research on effective targets. Currently, the contribution of epithelial-mesenchymal transition (EMT) to keloid overgrowth is appealing as a potential treatment target. High expression of mesenchymal markers, such as vimentin and fibronectin, and decreased expression of the epithelial marker E‐cadherin confirmed EMT phenomenon in keloids [6]. EMT refers to the biological process associated with the loss of polarity of epithelial cells and their transformation into mesenchymal cells with migratory and invasive characteristics [7]. It has been reported that inflammatory stimuli and a hypoxic microenvironment may induce the transition from keratinocytes to fibroblasts, facilitating the invasive growth of keloids [8].

Long noncoding RNAs (lncRNAs) are widely conserved sequences longer than 200 nucleotides without protein-coding capacity [9]. As an emerging class of regulatory RNAs, lncRNAs may participate in fundamental processes including cell cycle regulation, proliferation, apoptosis, self-renewal, metastasis and the DNA damage response through transcriptional or posttranscriptional regulation and thus give rise to diseases. Abnormally expressed lncRNAs play a key role in fibrosis [10] and different types of cancer, including breast cancer [11–13], lung cancer [14, 15], colorectal cancer [16, 17], esophageal squamous cell carcinoma [18], and cutaneous squamous cell carcinoma [19, 20].

The role of lncRNAs in normal skin and keloids is an emerging research topic. LncRNAs such as antidifferentiation noncoding RNA (ANCR) and terminal differentiation-induced noncoding RNA (TINCR) are critical in maintaining epidermal stability [21]. In addition to regulating normal skin formation, it has been reported that lncRNAs may influence the occurrence and development of keloids. LncRNAs are aberrantly expressed in keloid tissues compared with normal skin tissues, indicating that differentially expressed lncRNAs may participate in keloid formation [22]. Although the lncRNA expression profiles of keloids have been reported, few studies have concentrated on the physiological functions of lncRNAs. Zhu et al. [23] reported increased lncRNA-ATB expression in keloids, and revealed that lncRNA-ATB regulates the autocrine secretion of TGF-β2 in keloid fibroblasts by downregulating ZNF217 via miR-200c. Yuan et al*.* [24] identified the upregulation of lncRNA LINC01116 in keloids and demonstrated that LINC01116 accelerates the development of keloids by regulating the miR-203/SMAD5 axis. In addition, differential expression profiles of lncRNAs associated with the Hedgehog and Wnt pathways suggest a possible regulatory mechanism of lncRNAs in the occurrence and development of keloids [25, 26].

Many studies have confirmed the connection between lncRNAs and EMT [27–30] in multiple diseases, especially in tumors [31–35]. Chen et al. [36] reported that lncRNA HOXA11-AS upregulation promotes EMT by inhibiting miR-200b in non-small cell lung cancer (NSCLC) and predicts a poor prognosis. LncRNA NORAD was also reported to enhance invasion and EMT in NSCLC cells [37], while lncRNA H19 was found to facilitate the EMT and metastasis of esophageal cancer cells through the let-7c/STAT3/EZH2/β-catenin axis [38]. In addition, increased expression of lncRNA UCA1 plays a role in EMT occurrence by targeting miR-155 and can be used as survival prediction in cervical cancer [39]. Nevertheless, to date, no research has focused on EMT-related lncRNAs in keloids. Considering the role of EMT in the pathogenesis of keloids, this study made the first effort into detecting and analyzing EMT-related lncRNA expression profiles in keloids.

## Results

### RNA quality testing

**Fig. 1:**
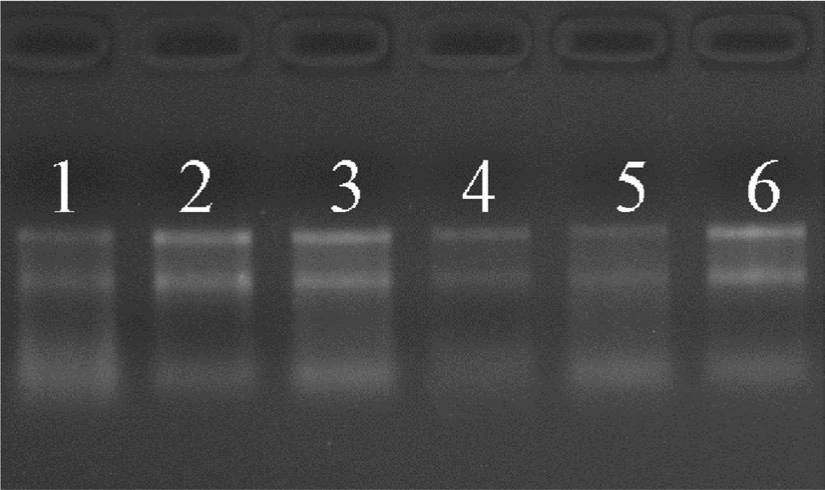
RNA integrity and gDNA contamination were assessed by denaturing agarose gel electrophoresis. Lanes 1–3 correspond to RNA samples of A1–C1, and lanes 4–6 represent RNA samples of A2–C2

The baseline data of the 3 patients included in the study are shown in Table 1. We extracted total RNA from keloids and adjacent normal skin and carried out quality testing by a NanoDrop ND-1000 spectrophotometer (Table 2). The A260/A280 ratios of RNAs extracted from the keloid and normal groups were between 1.8 and 2.1, and the A260/A230 ratios were < 1.8, indicating good-quality RNA samples. The imaging of the agarose gel after electrophoresis demonstrated fairly sharp and intense 18 s and 28 s bands, indicating the acceptable purity and integrity of RNA samples (Fig. 1).

**Table 1:** Baseline data of the included patients

Sample ID	Age (years)	Sex	Lesion location
A	21	Male	Cheek
B	67	Female	Abdomen
C	30	Female	Chest

**Table 2:** RNA quantification and quality assurance by NanoDrop ND-1000

Sample ID	OD260/280 ratio	OD260/230 ratio	Conc. (ng/μl)	Volume (μl)	Quantity (ng)	QC result (pass/fail)
A1	1.93	1.83	396.15	40	15,846.00	Pass
B1	1.93	1.95	296.42	30	8892.60	Pass
C1	1.97	1.98	644.77	40	25,790.80	Pass
A2	1.90	2.03	440.80	40	17,632.00	Pass
B2	1.92	2.21	298.85	30	8965.50	Pass
C2	1.92	1.87	435.73	40	17,429.20	Pass

A1–C1 refer to RNAs extracted from keloids, and A2–C2 refer to RNAs extracted from adjacent normal skin from the corresponding patients

A1–C1 refer to RNAs extracted from keloids, and A2–C2 refer to RNAs extracted from adjacent normal skin from the corresponding patients.

### Differentially expressed lncRNAs and mRNAs

A total of 773 EMT-related lncRNAs and 219 mRNAs were evaluated in the study, 11 lncRNAs and 16 mRNAs were identified to have differential expression between keloid tissue and normal skin tissue (fold change > 1.5, *P* < 0.05) (Tables 3 and 4). Among the detected lncRNAs, 7 were upregulated in keloid specimens, while 4 were downregulated. Regarding differentially expressed mRNAs, 12 were significantly upregulated, and 4 were significantly downregulated in keloids.

**Table 3:** Differentially expressed EMT-related lncRNAs in keloids compared with normal skin

Seq name	Gene name	Absolute fold change	Regulation
ENST00000539135	RP11-264F23.3	2.5210468	Up
uc001gch.1	AF268386	4.4642715	Up
AK055628	AK055628	56.9629769	Up
NR_033321	MIAT	5.7549102	Up
NR_024065	LINC00312	1.7928701	Up
TCONS_00002241	XLOC_000587	3.6222026	Up
ENST00000563754	RP11-95H3.1	3.3825798	Up
uc003djn.3	BC041347	1.6365448	Down
NR_004436	SNAR-A2	2.9651001	Down
NR_002605	DLEU1	2.1737057	Down
TCONS_00014246	XLOC_006329	1.7835828	Down

**Table 4:** Differentially expressed EMT-related mRNAs in keloids compared with normal skin

Seq name	Gene name	Absolute fold change	Regulation
NM_001111285	IGF1	2.6027441	Up
NM_006475	POSTN	27.6007449	Up
NM_013227	ACAN	7.4733116	Up
NM_005985	SNAI1	5.3111159	Up
NM_003118	SPARC	26.7924263	Up
NM_002211	ITGB1	3.4373866	Up
NM_003380	VIM	4.4300878	Up
NM_019554	S100A4	2.8472375	Up
NM_003239	TGFB3	7.59837	Up
uc003pan.1	MCM3	1.6558978	Up
NM_004126	GNG11	3.5309769	Up
ENST00000304636	COL3A1	29.5454592	Up
NM_020061	OPN1LW	4.2499062	Down
NM_024494	WNT2B	4.3460703	Down
NM_017588	WDR5	4.0202322	Down
NM_005238	ETS1	2.3434605	Down

**Fig. 2:**
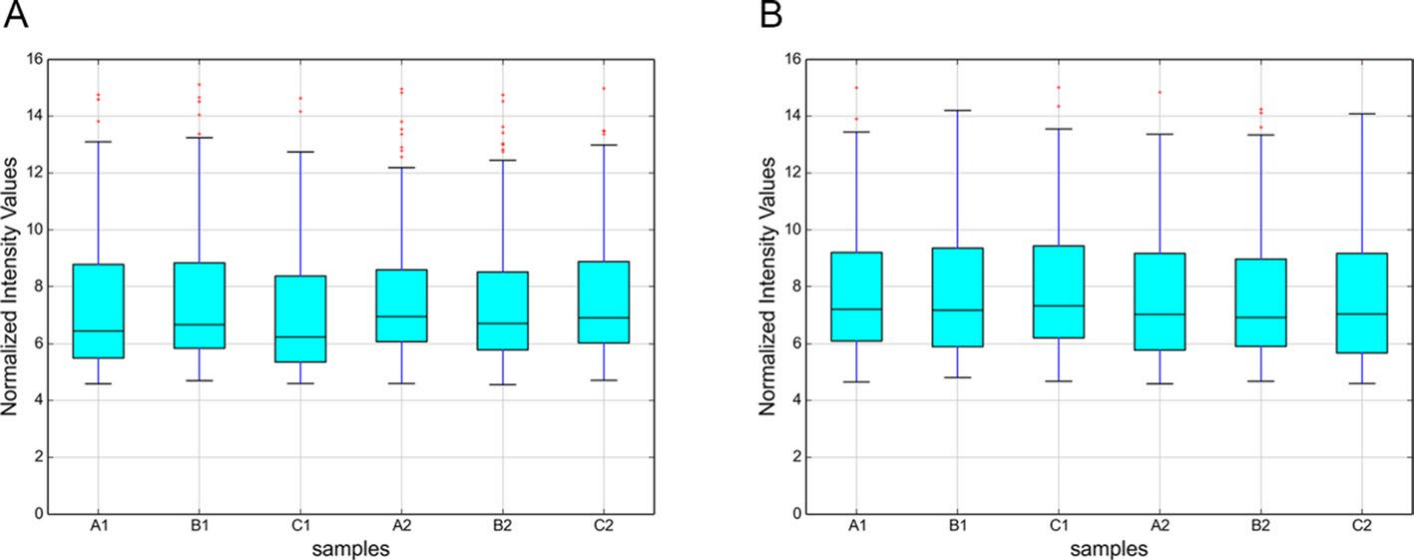
Box-whisker plots of lncRNAs (**A**) and mRNAs (**B**) in keloid specimens and normal skin specimens show similar distributions of intensities for all samples

**Fig. 3:**
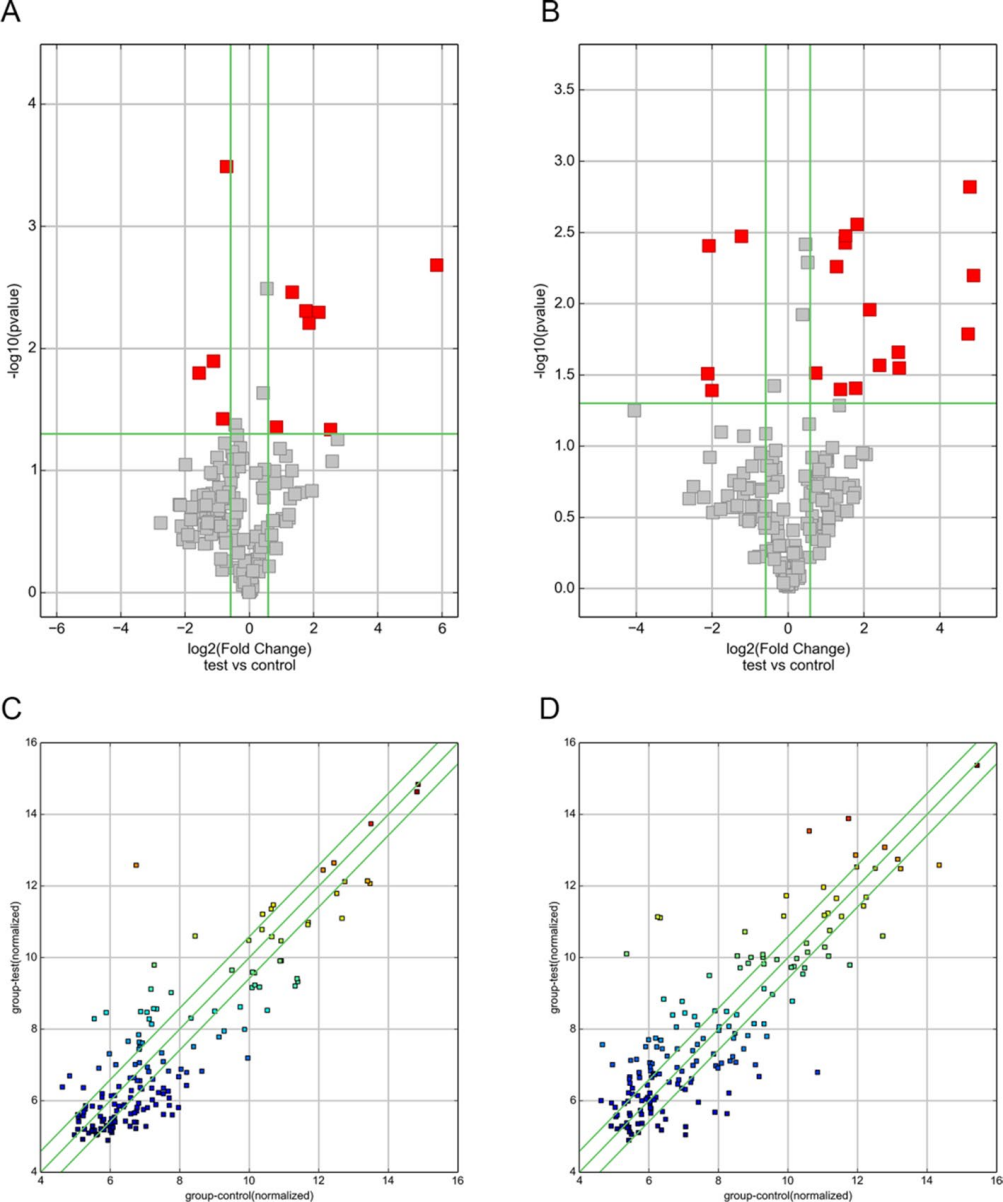
Expression profiles of EMT-related lncRNAs and mRNAs in keloid specimens and normal skin specimens. Volcano plots of lncRNA (**A**) and mRNA (**B**) expression profiles showing differential expression between keloid tissue and normal skin tissue. The horizontal green line represents a *P*-value of 0.05 and the vertical green lines correspond to 1.5-fold upregulation and downregulation. The red points represent differentially expressed RNAs with statistical significance. Scatter plots of lncRNAs (**C**) and mRNAs (**D**) are presented for the assessment of expression variation between keloid tissue and normal skin tissue. The values of the *x* and *y*-axes are the averaged normalized signal values (log2 scaled), and the default fold change given is 1.5

**Fig. 4:**
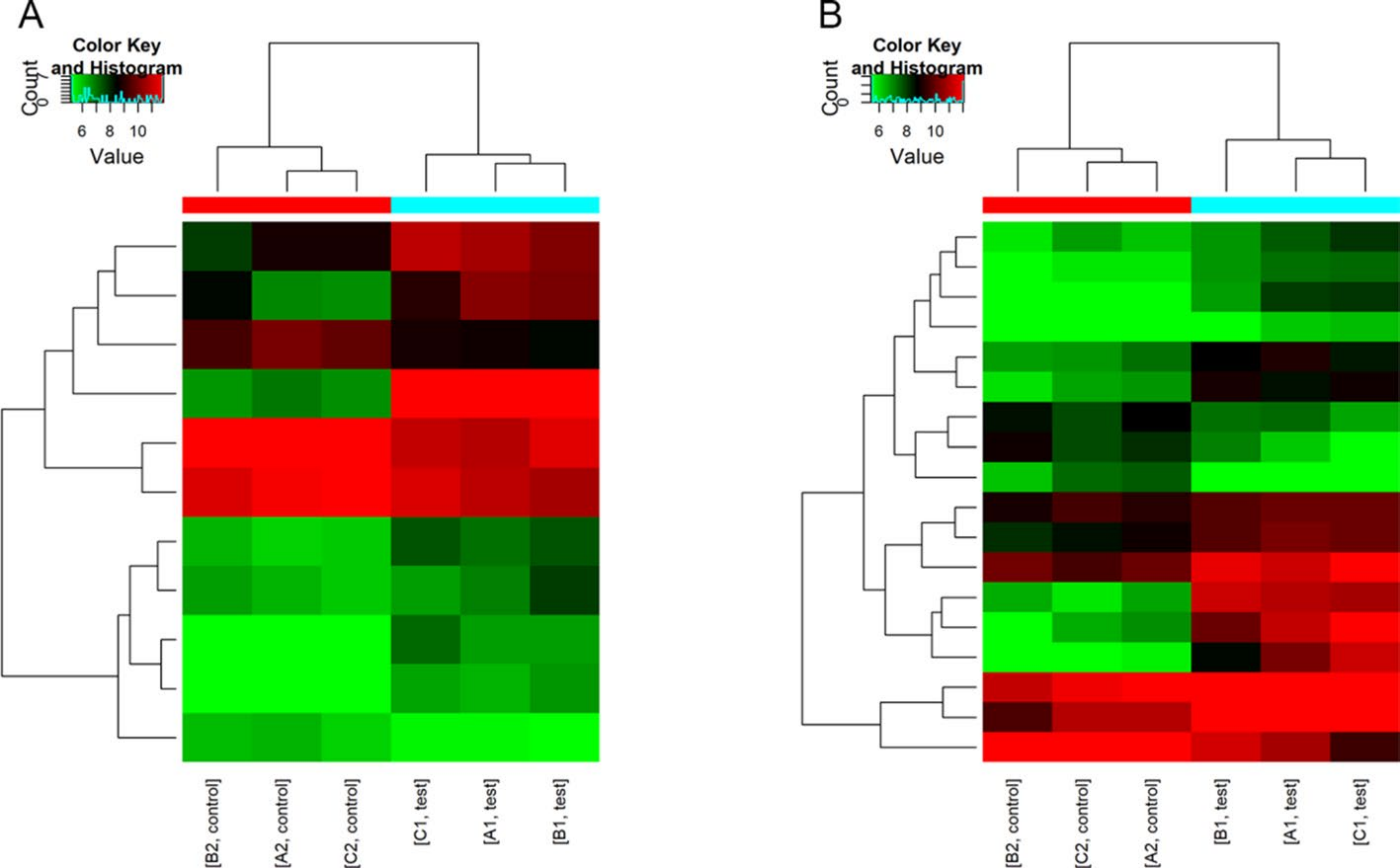
Hierarchical clustering of lncRNAs (**A**) and mRNAs (**B**) classifies samples into groups according to expression level. Each column corresponds to a sample, and each row stands for a gene. Red and green colors represent high and low expression of genes, respectively

To compare the intensity distribution of 6 samples, we used box-whisker plots, which suggested that the data distribution from 6 chips was similar (Fig. 2). Variations in EMT-related lncRNAs and mRNA expression among specimens are shown in volcano plots and scatter plots (Fig. 3). Based on the microarray results, hierarchical clustering was performed to infer the relationship among specimens and demonstrated distinguishable lncRNA and mRNA expression profiles between keloid tissue and normal skin tissue (Fig. 4).

By quantifying lncRNAs at the transcript level and their potential target genes at both the gene and transcript levels in parallel, microarray analysis helped to establish connections between the regulatory mechanisms and biological functions of the lncRNAs. Five pairs of predicted lncRNA–mRNA were listed as examples (Table 5). More information could be found in the additional file (see Additional file 1).

**Table 5:** Predicted regulation of genes by lncRNAs

Gene symbol	Fold change	Regulation	Genomic relationship	Potential mechanism	mRNA symbol
RP11-264F23.3	2.521047	Up	Overlapping	Neighboring	CCND2
AF268386	4.464272	Up	Upstream	Enhancer	DDR2
XLOC_000587	3.622203	Up	Upstream	Enhancer	ENAH
MT1CP	1.344673	Up	Downstream	Enhancer	MT1B
XLOC_006329	1.783583	Down	Upstream	Enhancer	PDGFA

### Gene Ontology (GO) analysis

**Fig. 5:**
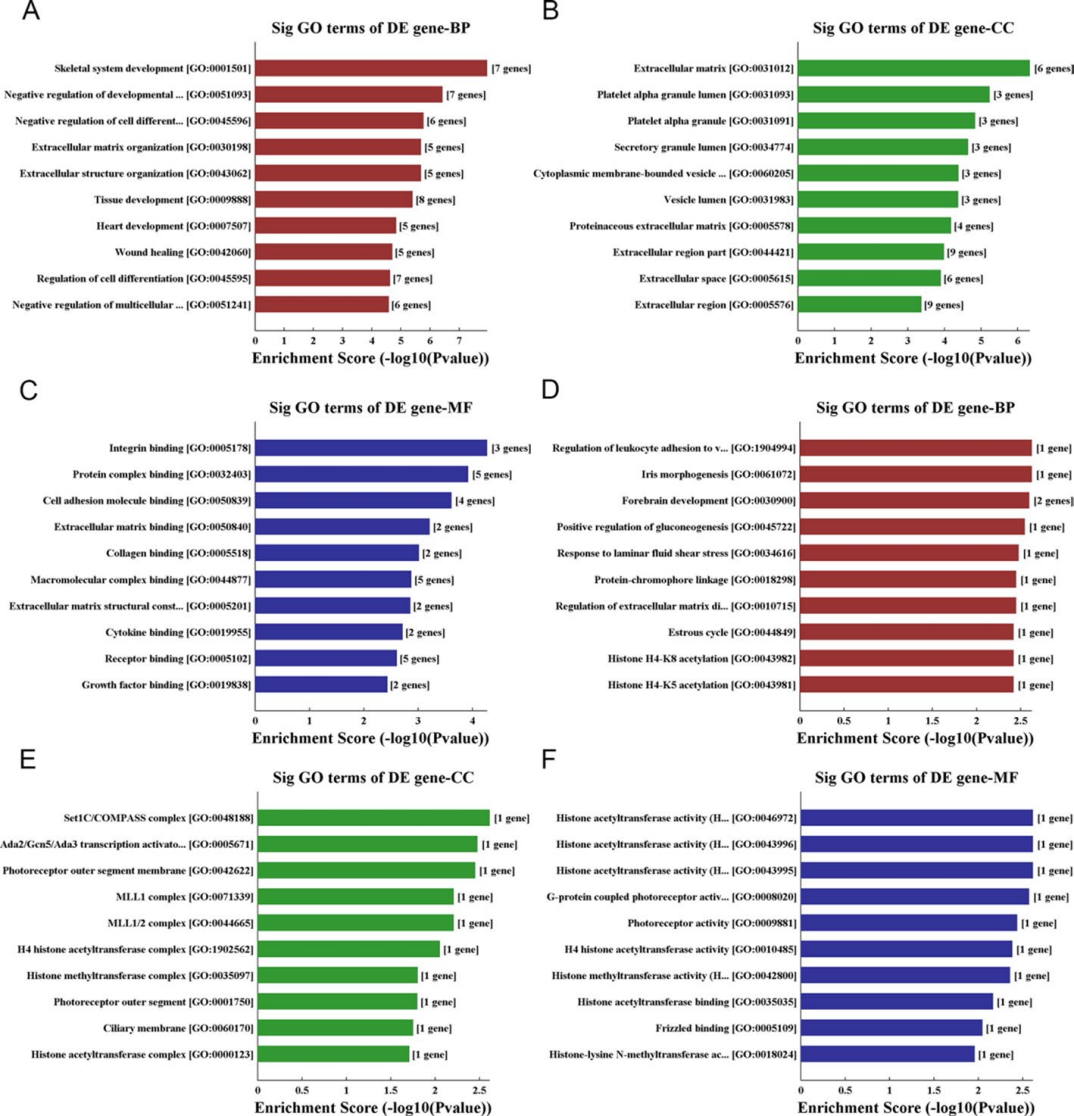
GO analysis of differentially expressed coding gene transcripts. The most highly enriched GO terms for the upregulated transcripts in keloids in terms of biological processes (**A**), cellular components (**B**), and molecular functions (**C**). The most highly enriched GO terms for the downregulated transcripts in terms of biological processes (**D**), cellular components (**E**), and molecular functions (**F**)

Utilizing GO, the de facto standard in describing gene functions, we carried out functional annotation and enrichment analysis of differentially expressed transcripts. We found that in upregulated transcripts from keloids, the most enriched GO terms were single-organism cellular processes (biological process), the intracellular part (cellular component), and protein binding (molecular function) (Fig. 5A–C). For downregulated transcripts, the most enriched GO terms were the positive regulation of cellular processes (biological process), the Set1C/COMPASS complex (cellular component), and histone acetyltransferase activity (molecular function) (Fig. 5D–F).

### Kyoto Encyclopedia of Genes and Genomes (KEGG) analysis

**Fig. 6:**
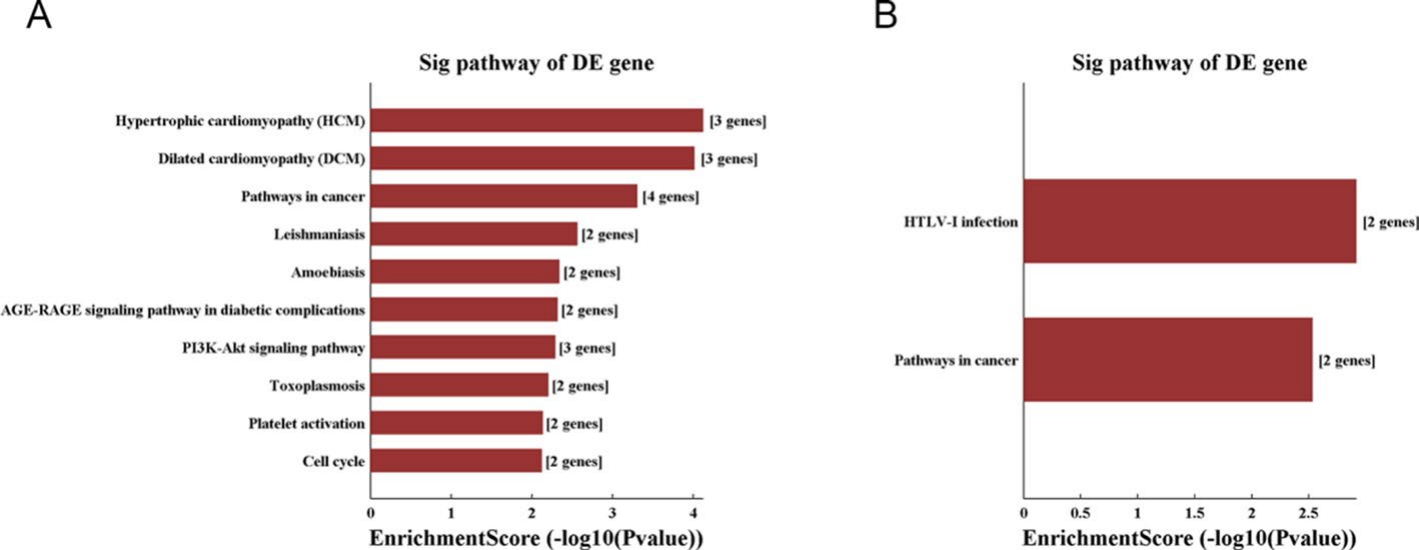
Pathway analysis. Pathways related to upregulated (**A**) and downregulated (**B**) transcripts in keloids

KEGG analysis helped us to map lncRNAs to pathways, and this analysis demonstrated a total of 10 pathways related to upregulated transcripts in keloids (Fig. 6A). The top 3 most enriched networks were hypertrophic cardiomyopathy (HCM), dilated cardiomyopathy (DCM) and pathways in cancer. Moreover, 2 pathways, corresponding to HTLV-1 infection and pathways in cancer, were found to be related to downregulated transcripts in keloids (Fig. 6B).

### Validation of the microarray finding by qRT-PCR

**Fig. 7:**
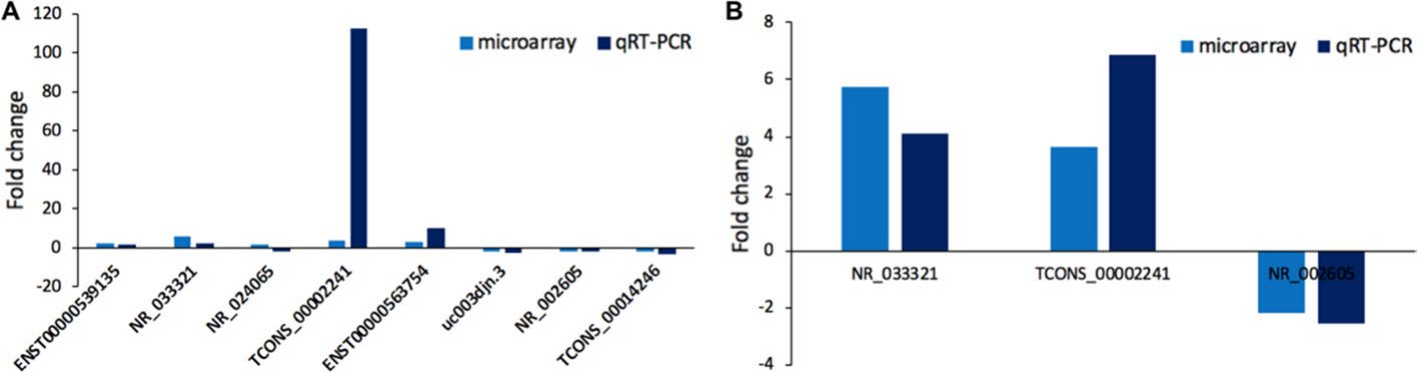
Relative expression of candidate lncRNAs detected by qRT-PCR. **A** Comparison of the fold changes of 8 lncRNAs between microarray and qRT-PCR results in original specimens. **B** Validation of 3 chosen lncRNAs by qRT-PCR in an enlarged sample set

To validate the microarray results, we performed qRT-PCR of 5 upregulated lncRNAs (ENST00000539135, NR_033321, NR_024065, TCONS_00002241 and ENST00000563754) and 3 downregulated lncRNAs (uc003djn.3, NR_002605 and TCONS_00014246) in the original specimens (Fig. 7A). NR_033321, TCONS_00002241, ENST00000563754, and NR_002605 were proven to have significant changes between the original keloid specimens and normal skin specimens. Among these verified lncRNAs, TCONS_00002241 demonstrated the most apparent variation with fold change up to 112.53.

A total of 12 pairs of keloid specimens and adjacent normal skin specimens were then tested by qRT-PCR to increase the sample size, and the expression of NR_033321, TCONS_00002241, and NR_002605 was detected (Fig. 7B). All these lncRNAs were significantly differentially expressed. Consistent with the validation results in the original specimens, TCONS_00002241 presented the most evident fold change (6.88).

## Discussion

This study utilized an EMT-specific microarray and bioinformatics analysis to evaluate EMT-related lncRNAs, mRNAs, and involved signaling pathways in keloids. The results of the microarray are summarized in Tables 3 and 4 and were verified by qRT-PCR in a larger set of samples to guarantee reliability. Compared to the use of qRT-PCR alone to assess certain lncRNAs commonly investigated in cancer, microarray analysis provides a more macroscopic differential expression profile, helping to identify novel lncRNAs involved in the pathogenesis of keloid diseases. Because the functions of many lncRNAs are still elusive while corresponding mRNAs are better understood, we conducted GO and KEGG analyses of target gene mRNAs to analyze the regulatory mechanism and biological function of lncRNAs in keloids. GO analysis showed that the functions of these mRNAs were related to the extracellular matrix, protein binding, the positive regulation of cellular process, the Set1C/COMPASS complex, and histone acetyltransferase activity, suggesting that the differentially expressed lncRNAs and mRNAs play a role in the development of tumors. Following GO analysis, the subsequent KEGG analysis demonstrated that the mRNAs are involved in pathways in cancer. Considering that keloids are benign lesions characterized by invasive growth, it is reasonable that pathways in cancer are recognized to be differentially enriched between keloids and normal skin tissue.

Notably, XLOC_000587, the corresponding gene of TCONS_00002241, is located within 300 kb upstream of ENAH and may be an enhancer of ENAH. ENAH is an actin regulatory protein involved in cell motility and adhesion. Extensive studies have demonstrated upregulation of ENAH in many tumors, including breast carcinoma [40], cervical carcinoma [41], colorectal carcinoma [42], and hepatocellular carcinoma [43]. Notably, the expression of ENAH is correlated with vascular invasion, tumor grade, and unfavorable prognosis, which suggests that ENAH might participate in carcinogenesis and tumor progression [44–46]. Chen et al. [45] reported that EMT may be involved in the role of ENAH in gastric cancer cell proliferation and metastasis. EMT is regulated by a complex network including the TGF-β, Wnt, and Notch signaling pathways [47], while ENAH has been demonstrated to function as a transcriptional target of the Wnt/β-catenin pathway and a novel connection between the Wnt/β-catenin and Notch signaling cascades [48]. Therefore, we speculate that XLOC_000587 in keloids may further regulate related signaling pathways by increasing the expression of ENAH to promote EMT and the invasive development of keloids. Elucidating the mechanism by which XLOC_000587 participates in EMT is expected to provide new insights into keloid treatment.

In addition, AF268386 was predicted to be an enhancer of discoidin domain receptor 2 (DDR2), a tyrosine kinase receptor activated by collagen [49]. As a recognized critical regulator of EMT, DDR2 upregulation has been confirmed in many tumor cell lines and takes part in the interaction with the extracellular matrix, the regulation of cell motility and adhesion, and metastatic spreading [49–52]. Toy et al. [53] reported that overexpressed DDR2 in triple-negative breast cancer may indicate carcinoma with worse prognosis. Moreover, Ren et al. [54] reported that DDR2 expression was closely correlated with the expression of the hypoxic marker HIF-1α in breast cancer specimens, and hypoxia treatment could induce the expression of DDR2 in human breast cancer cell lines. Regarding wound healing, DDR2 is capable of promoting proliferation, collagen I secretion and MMP2 production in skin fibroblasts, and a delay in wound healing was observed in DDR2 null mice [55]. According to the role of DDR2 in wound healing and metastatic spreading and the hypoxic microenvironment in keloids, we hypothesize that targeting AF268386 or the corresponding lncRNA AF268386 could be a potential therapeutic approach for keloids treatment. In view of the limited research findings, more studies concerning these candidate lncRNAs remain to be carried out to uncover underlying pathways, establish lncRNA–miRNA–mRNA molecular network and clarify the exact functions in the occurrence and development of keloids.

## Conclusion

This study identified and verified the differential expression of EMT-related lncRNAs in keloids, indicating that lncRNAs are likely to be involved in EMT and the overgrowth of keloids. In particular, lncRNA XLOC_000587 may participate in cell proliferation and migration by enhancing the expression of ENAH, and AF268386 may accelerate the invasive development of keloids by upregulating DDR2. The differentially expressed lncRNAs will hopefully provide novel insights into the pathogenesis of keloid diseases by multiple mechanisms such as signal transduction and competing endogenous RNA (ceRNA) networks.

## Methods

### Patients and specimens

The study complied with the Declaration of Helsinki of the World Medical Association and was approved by the Ethics Committee of The First Affiliated Hospital, Zhejiang University School of Medicine. All 12 patients were fully informed of the aim and protocol of the study, gave written informed consent, and provided excised keloids and adjacent normal skin for scientific research and publication. None of the patients had previously undergone radiotherapy or drug therapy. The keloids had clinical characteristics of overgrowth beyond the original wound boundaries and were confirmed by pathology. Tissues were frozen in liquid nitrogen and stored at − 80 °C immediately after excision.

### RNA extraction and quality control

Total RNA was extracted from frozen specimens using TRIzol reagent (Invitrogen, Carlsbad, CA, USA) following the manufacturer's instructions (Invitrogen). The RNA quality was assessed by NanoDrop ND-1000 (Thermo Fisher Scientific, Waltham, MA, USA), and RNA integrity was assessed by denaturing agarose gel electrophoresis.

### RNA labeling and array hybridization

Keloid and normal skin specimens from 3 randomly selected patients were tested by microarray profiling. The chip used was the lncPath Human EMT Array (8 × 15 K) (ArrayStar, Rockville, MD, USA). Microarray analysis was performed by Kangchen Biology Engineering Co., Ltd., (Shanghai, China) according to the manufacturer’s protocol (Arraystar). LncRNAs/mRNAs were purified from total RNA after removal of rRNA (Arraystar rRNA removal kit). Then, each sample was amplified and transcribed into fluorescent cRNA along the entire length of the transcripts without 3′ bias utilizing a random priming method (Arraystar Flash RNA Labeling Kit, Arraystar). The labeled cRNAs were purified by RNeasy Mini Kit (Qiagen, Germany). The concentration and specific activity of the labeled cRNAs (pmol Cy3/μg cRNA) were measured by NanoDrop ND-1000. One microgram of each labeled cRNA was fragmented by adding 5 μl 10 × Blocking Agent and 1 μl 25 × Fragmentation Buffer and then heating the mixture at 60 °C for 30 min. Finally, 25 μl 2 × GE Hybridization buffer was added to dilute the labeled cRNA. Fifty microliters of hybridization solution was dispensed into the gasket slide and assembled into the lncRNA expression microarray slide. The slides were incubated for 17 h at 65 °C in an Agilent Hybridization Oven. The hybridized arrays were washed, fixed and scanned using the Agilent Scanner G2505C.

### Data analysis

Differentially expressed lncRNAs/mRNAs were defined as those with a fold change ≥ 1.5 (upregulated or downregulated) combined with *P*-value < 0.05. Agilent Feature Extraction software (version 11.0.1.1) was used to analyze acquired array images. Quantile normalization and subsequent data processing were performed using the R software limma package. Differentially expressed lncRNAs/mRNAs with statistical significance between two groups were identified through volcano plot filtering. Differentially expressed lncRNAs/mRNAs between two samples were identified through fold change filtering. Hierarchical clustering was performed to show the distinguishable lncRNA/mRNA expression pattern among samples.

Based on the Gene Ontology (GO) database (http://www.geneontology.org), the differential transcripts underwent enrichment analysis of gene function via GO analysis, which indicates enriched terms in the categories of biological process, molecular function and cellular component. Pathway analysis of identified differentially expressed genes was performed using the Kyoto Encyclopedia of Genes and Genomes (KEGG) database (http://www.genome.jp/kegg/).

### Real-time quantitative polymerase chain reaction (qRT-PCR)

Reverse transcription of extracted RNA was performed utilizing SuperScript™ III reverse transcriptase (Invitrogen) following the manufacturer’s instructions. qRT-PCR was conducted using the ethidium bromide staining method with ViiA 7 real-time PCR system (Applied Biosystems) following the manufacturer’s protocols.

Five significantly upregulated (ENST00000539135, NR_033321, NR_024065, TCONS_00002241 and ENST00000563754) and 3 downregulated lncRNAs (uc003djn.3, NR_002605 and TCONS_00014246) were selected for qRT-PCR validation in the original specimens. NR_033321, NR_002605 and TCONS_00002241 were further detected in a larger set of samples including 12 pairs of keloid tissues and adjacent normal skin tissues. β-Actin was used as endogenous control. The primer sequences were as follows:

β-actin:

Forward: 5′ GTGGCCGAGGACTTTGATTG3′

Reverse: 5′ CCTGTAACAACGCATCTCATATT3′

ENST00000539135:

Forward: 5′ AGCCAAGAAACGGTCCAGA 3′

Reverse: 5′ AGCACAGGTAGAGATCAGGAGG 3′

NR_033321:

Forward: 5′ GACCCGAGTTGGAGGCATCT 3′

Reverse: 5′ TGTGGTTGTGAACGTCCTGAAG3′

NR_024065:

Forward: 5′ GCTATTAGAGGGTGTTGCTGAC3′

Reverse: 5′ TGTATTGTGGCTATGTGGGAG 3′

TCONS_00002241:

Forward: 5′ CACATTTACAGACTACAGAGCC 3′

Reverse: 5′ CTGACCACACCATCAGCAAC 3′

ENST00000563754:

Forward: 5′ CATTCCTGAGGCTGTTCGT 3′

Reverse: 5′ GAAAATGTCTCTGAGAACCCAT 3′

uc003djn.3:

Forward: 5′ AGCACAGCCTGAAACCCAA 3′

Reverse: 5′ AGGAAGCATCTCACCCTCCT 3′

NR_002605:

Forward: 5′ CCTTGTATATGGATACACGTGC 3′

Reverse: 5′ TCCTTTCTGGTAGAATCACTGG 3′

TCONS_00014246:

Forward: 5′ CCGAAAAGCGAACAGTCCA 3′

Reverse: 5′ CGTGTTTGGTGAGTTCGGTT 3′.

The reaction conditions for PCR were as follows: 95 °C for 10 min, then 40 PCR cycles at 95 °C for 10 s and 60 °C for 60 s. Products were slowly heated from 60 to 99 °C after amplification, and melting curve was established to test the specificity of PCR products. The corrected relative expression level was acquired by the following calculation, the concentration of the target gene divided by the concentration of the housekeeping gene.

### Statistical analysis

All data were analyzed utilizing SPSS (version 18.0; SPSS, Inc., Chicago, IL, USA). The Student’s *t*-test was performed to evaluate the differences between distinct groups, and one-way ANOVA and SNK methods were used for comparisons among groups. A *P*-value < 0.05 was considered significant.

## Supplementary Information

**Additional file 1.** Predicted regulation of 100 pairs of lncRNA–mRNA.

## Data Availability

The microarray datasets in the present study are available on GEO with accession number GSE182192.

## Abbreviations

EMT: Epithelial-mesenchymal transition
lncRNA: Long noncoding RNA
qRT-PCR: Real-time quantitative polymerase chain reaction
GO: Gene Ontology
KEGG: Kyoto Encyclopedia of Genes and Genomes
TGF-β: Transforming growth factor-β
CTGF: Connective tissue growth factor
VEGF: Vascular endothelial growth factor
ANCR: Antidifferentiation noncoding RNA
TINCR: Terminal differentiation-induced noncoding RNA
NSCLC: Non-small cell lung cancer
HCM: Hypertrophic cardiomyopathy
DCM: Dilated cardiomyopathy
DDR2: Discoidin domain receptor 2

## References

[1] Jagdeo J, Kerby E, Glass DA (2021). Keloids are large, firm, raised scars that occur after skin injury. JAMA Dermatol.

[2] Berman B, Maderal A, Raphael B (2017). Keloids and hypertrophic scars: pathophysiology, classification, and treatment. Dermatol Surg.

[3] Naik PP (2021). Review on novel targets and therapies for Keloids. Clin Exp Dermatol.

[4] Hedayatyanfard K, Haddadi N-S, Ziai SA, Karim H, Niazi F, Steckelings UM (2020). The renin-angiotensin system in cutaneous hypertrophic scar and keloid formation. Exp Dermatol.

[5] Stevenson AW, Deng Z, Allahham A, Prele CM, Wood FM, Fear MW (2021). The epigenetics of keloids. Exp Dermatol.

[6] Ma XY, Chen J, Xu B, Long X, Qin H, Zhao RC (2015). Keloid-derived keratinocytes acquire a fibroblast-like appearance and an enhanced invasive capacity in a hypoxic microenvironment in vitro. Int J Mol Med.

[7] Yuan FL, Sun ZL, Feng Y, Liu SY, Du Y, Yu S (2019). Epithelial-mesenchymal transition in the formation of hypertrophic scars and keloids. J Cell Physiol.

[8] Kuwahara H, Tosa M, Egawa S, Murakami M, Mohammad G, Ogawa R (2016). Examination of epithelial mesenchymal transition in keloid tissues and possibility of keloid therapy target. Plast Reconstr Surg Glob Open.

[9] Borkiewicz L, Kalafut J, Dudziak K, Przybyszewska-Podstawka A, Telejko I (2021). Decoding LncRNAs. Cancers.

[10] Xu Q, Cheng D, Liu Y, Pan H, Li G, Li P (2021). LncRNA-ATB regulates epithelial-mesenchymal transition progression in pulmonary fibrosis via sponging miR-29b-2-5p and miR-34c-3p. J Cell Mol Med.

[11] Li Z, Qian Z, Chen F, Jiang S, Meng L, Chen J (2021). Identification of key lncRNA-mRNA pairs and functional lncRNAs in breast cancer by integrative analysis of TCGA data. Front Genet.

[12] Fang K, Xu Z-J, Jiang S-X, Tang D-S, Yan C-S, Deng Y-Y (2021). lncRNA FGD5-AS1 promotes breast cancer progression by regulating the hsa-miR-195-5p/NUAK2 axis. Mol Med Rep.

[13] Liu J, Zhao G, Liu X-L, Zhang G, Zhao S-Q, Zhang S-L (2021). Progress of non-coding RNAs in triple-negative breast cancer. Life Sci.

[14] Wan Y, Yao D, Fang F, Wang Y, Wu G, Qian Y (2021). LncRNA WT1-AS downregulates lncRNA UCA1 to suppress non-small cell lung cancer and predicts poor survival. BMC Cancer.

[15] Chen Y, Zhou X, Huang C, Li L, Qin Y, Tian Z (2021). LncRNA PART1 promotes cell proliferation and progression in non-small-cell lung cancer cells via sponging miR-17-5p. J Cell Biochem.

[16] Wu Y, Cong L, Chen W, Wang X, Qiu F (2021). lncRNA LINC00963 downregulation regulates colorectal cancer tumorigenesis and progression via the miR-10b/FGF13 axis. Mol Med Rep.

[17] Wang M, Zhang Z, Pan D, Xin Z, Bu F, Zhang Y (2021). Biosci Rep.

[18] Pan Q, Li B, Zhang J, Du X, Gu D (2021). LncRNA THAP9-AS1 accelerates cell growth of esophageal squamous cell carcinoma through sponging miR-335–5p to regulate SGMS2. Pathol Res Pract.

[19] Lu D, Sun L, Li Z, Mu Z (2021). lncRNA EZR-AS1 knockdown represses proliferation, migration and invasion of cSCC via the PI3K/AKT signaling pathway. Mol Med Rep.

[20] Xu Y, Dong Y, Deng Y, Qi Q, Wu M, Liang H (2021). Identifying an lncRNA-related ceRNA network to reveal novel targets for a cutaneous squamous cell carcinoma. Biology.

[21] Hombach S, Kretz M (2013). The non-coding skin: exploring the roles of long non-coding RNAs in epidermal homeostasis and disease. BioEssays.

[22] Liang X, Ma L, Long X, Wang X (2015). LncRNA expression profiles and validation in keloid and normal skin tissue. Int J Oncol.

[23] Zhu H-Y, Bai W-D, Li C, Zheng Z, Guan H, Liu J-Q (2016). Knockdown of IncRNA-ATB suppresses autocrine secretion of TGF-beta 2 by targeting ZNF217 via miR-200c in keloid fibroblasts. Sci Rep.

[24] Yuan W, Sun H, Yu L (2021). Long non-coding RNA LINC01116 accelerates the progression of keloid formation by regulating miR-203/SMAD5 axis. Burns.

[25] Huang H, Fu S, Liu D (2018). Detection and analysis of the hedgehog signaling pathway-related long non-coding RNA (IncRNAs) expression profiles in keloid. Med Sci Monit.

[26] Sun X-j, Wang Q, Guo B, Liu X-y, Wang B (2017). Identification of skin-related lncRNAs as potential biomarkers that involved in Wnt pathways in keloids. Oncotarget.

[27] Zhou J, Jiang H (2019). Livin is involved in TGF-beta 1-induced renal tubular epithelial-mesenchymal transition through lncRNA-ATB. Ann Transl Med.

[28] Peng P-H, Lai JC-Y, Hsu K-W, Wu K-J (2020). Hypoxia-induced lncRNA RP11-390F43 promotes epithelial-mesenchymal transition (EMT) and metastasis through upregulating EMT regulators. Cancer Lett.

[29] Liu Y, Li Y, Xu Q, Yao W, Wu Q, Yuan J (2018). Long non-coding RNA-ATB promotes EMT during silica-induced pulmonary fibrosis by competitively binding miR-200c. Biochim Biophys Acta Mol Basis Dis.

[30] Fan Y, Zhao X, Ma J, Yang L (2021). LncRNA GAS5 competitively combined with miR-21 regulates PTEN and influences EMT of peritoneal mesothelial cells via Wnt/beta-catenin signaling pathway. Front Physiol.

[31] Chen P-P, Zhang Z-S, Wu J-C, Zheng J-F, Lin F (2021). LncRNA SNHG12 promotes proliferation and epithelial mesenchymal transition in hepatocellular carcinoma through targeting HEG1 via miR-516a-5p. Cell Signal.

[32] Expression of concern: long non coding RNA FAM3D-AS1 inhibits development of colorectal cancer through NF-kB signaling pathway. Biosci Rep. 2021;41(4). 10.1042/BSR-20190724_EOC PMC8026816 33825868

[33] He L, Yang H, Zhu X-L, Zhang Y, Lv K (2021). Knockdown of long non-coding RNA SLC8A1-AS1 attenuates cell invasion and migration in glioma via suppression of Wnt/beta-catenin signaling pathways. Brain Res Bull.

[34] Su Y, Zhou L, Yu Q, Lu J, Liu W (2021). Long non-coding RNA LOC648987 promotes proliferation and metastasis of renal cell carcinoma by regulating epithelial-mesenchymal transition. Technol Cancer Res Treat.

[35] Sun Y, Gao X, Li P, Song L, Shi L (2021). LncRNA ZFAS1, as a poor prognostic indicator, promotes cell proliferation and epithelial-mesenchymal transition in endometrial carcinoma. Pers Med.

[36] Chen JH, Zhou LY, Xu S, Zheng YL, Wan YF, Hu CP (2017). Overexpression of lncRNA HOXA11-AS promotes cell epithelial-mesenchymal transition by repressing miR-200b in non-small cell lung cancer. Cancer Cell Int.

[37] Geng QQ, Li ZB, Li XT, Wu YH, Chen NZ (2021). LncRNA NORAD, sponging miR-363-3p, promotes invasion and EMT by upregulating PEAK1 and activating the ERK signaling pathway in NSCLC cells. J Bioenerg Biomembr.

[38] Chen M-J, Deng J, Chen C, Hu W, Yuan Y-C, Xia Z-K (2019). LncRNA H19 promotes epithelial mesenchymal transition and metastasis of esophageal cancer via STAT3/EZH2 axis. Int J Biochem Cell Biol.

[39] Yang TJ, Wang L, Zhang Y, Zheng JD, Liu L (2020). LncRNA UCA1 regulates cervical cancer surviva and EMT occurrence by targeting miR-155. Eur Rev Med Pharmacol Sci.

[40] Di Modugno F, Mottolese M, Di Benedetto A, Conidi A, Novelli F, Perracchio L (2006). The cytoskeleton regulatory protein hMena (ENAH) is overexpressed in human benign breast lesions with high risk of transformation and human epidermal growth factor receptor-2-positive/hormonal receptor-negative tumors. Clin Cancer Res.

[41] Gurzu S, Jung I, Prantner I, Chira L, Ember I (2009). The immunohistochemical aspects of protein Mena in cervical lesions. Rom J Morphol Embryol.

[42] Toyoda A, Kawana H, Azuhata K, Yu J, Omata A, Kishi H (2009). Aberrant expression of human ortholog of mammalian enabled (hMena) in human colorectal carcinomas: Implications for its role in tumor progression. Int J Oncol.

[43] Hu K, Wang J, Yao Z, Liu B, Lin Y, Liu L (2014). Expression of cytoskeleton regulatory protein Mena in human hepatocellular carcinoma and its prognostic significance. Med Oncol.

[44] Gurzu S, Krause M, Ember I, Azamfirei L, Gobel G, Feher K (2012). Mena, a new available marker in tumors of salivary glands?. Eur J Histochem.

[45] Chen D, Xu L, Li X, Chu Y, Jiang M, Xu B (2018). Enah overexpression is correlated with poor survival and aggressive phenotype in gastric cancer. Cell Death Dis.

[46] Wang D-D, Jin Q, Wang L-L, Han S-F, Chen Y-B, Sun G-D (2017). The significance of ENAH in carcinogenesis and prognosis in gastric cancer. Oncotarget.

[47] Yang J, Weinberg RA (2008). Epithelial-mesenchymal transition: at the crossroads of development and tumor metastasis. Dev Cell.

[48] Najafov A, Seker T, Even I, Hoxhaj G, Selvi O, Ozel DE (2012). MENA is a transcriptional target of the Wnt/beta-catenin pathway. PLoS ONE.

[49] Leitinger B (2003). Molecular analysis of collagen binding by the human discoidin domain receptors, DDR1 and DDR2—identification of collagen binding sites in DDR2. J Biol Chem.

[50] Badiola I, Villace P, Basaldua I, Olaso E (2011). Downregulation of discoidin domain receptor 2 in A375 human melanoma cells reduces its experimental liver metastasis ability. Oncol Rep.

[51] Walsh LA, Nawshad A, Medici D (2011). Discoidin domain receptor 2 is a critical regulator of epithelial-mesenchymal transition. Matrix Biol.

[52] Wang Y-G, Xu L, Jia R-R, Wu Q, Wang T, Wei J (2016). DDR2 induces gastric cancer cell activities via activating mTORC2 signaling and is associated with clinicopathological characteristics of gastric cancer. Dig Dis Sci.

[53] Toy KA, Valiathan RR, Nunez F, Kidwell KM, Gonzalez ME, Fridman R (2015). Tyrosine kinase discoidin domain receptors DDR1 and DDR2 are coordinately deregulated in triple-negative breast cancer. Breast Cancer Res Treat.

[54] Ren T, Zhang W, Liu X, Zhao H, Zhang J, Zhang J (2014). Discoidin domain receptor 2 (DDR2) promotes breast cancer cell metastasis and the mechanism implicates epithelial-mesenchymal transition programme under hypoxia. J Pathol.

[55] Olaso E, Labrador JP, Wang LH, Ikeda K, Eng FJ, Klein R (2002). Discoidin domain receptor 2 regulates fibroblast proliferation and migration through the extracellular matrix in association with transcriptional activation of matrix metalloproteinase-2. J Biol Chem.

