# Correction: Detection and analysis of long noncoding RNA expression profiles related to epithelial–mesenchymal transition in keloids

**DOI:** 10.1186/s12938-023-01149-0

**Published:** 2023-08-30

**Authors:** Zhixiong Chen, Xi Chu, Jinghong Xu

**Affiliations:** 1 Department of Plastic Surgery, The First Affiliated Hospital, Zhejiang University, School of Medicine, Hangzhou, 310003 People’s Republic of China; 2 Zhejiang University School of Medicine, Hangzhou, 310000 People’s Republic of China

**Correction** **:** **BioMedical Engineering OnLine (2022) 21:2** **https://doi.org/10.1186/s12938-022-00976-x**

Following publication of the original article [1], In the Funding of this article the grant number relating to Zhejiang Provincial Natural Science Foundation of China was incorrectly given as Q21H150018 and should have been LQ21H150006.

The original article has been corrected.
